# Utility of a Modified Oropharyngeal Airway for Performing Tracheal Intubation Using a Fiberoptic Bronchoscope and Video Stylet: A Randomized Crossover Trial Using a Manikin

**DOI:** 10.1155/2020/3017297

**Published:** 2020-10-29

**Authors:** Jang Hee Lee, Ji Ung Na, Dong Hyuk Shin, Pil Cho Choi, Sang O Park, Won Jae Kim, Sang Kuk Han

**Affiliations:** ^1^Department of Emergency Medicine, Kangbuk Samsung Hospital, Sungkyunkwan University School of Medicine, Seoul, Republic of Korea; ^2^Department of Emergency Medicine, School of Medicine, Konkuk University, Konkuk University Medical Centre, Seoul, Republic of Korea; ^3^Department of Emergency Medicine, Kangbuk Samsung Hospital, Seoul, Republic of Korea

## Abstract

**Purpose:**

The purpose of this study was to assess if a modified airway (MA), developed by the authors, would act as a guide and improve the performance of intubation when used with a video stylet (VS) or fiberoptic bronchoscope (FOB) for endotracheal intubation.

**Methods:**

This randomized crossover simulation study using manikins was conducted with 36 novice operators. Time to complete intubation, time to see the glottis, and success rate of intubation of each device were measured and compared with or without use of MA.

**Results:**

For intubation using FOB with MA, the median time to complete intubation significantly reduced from 46 to 31 seconds with a medium effect size (*p*=0.004, *r* = 0.483), and the median time to see the glottis significantly reduced from 7 to 5 seconds with a medium effect size (*p*=0.032, *r* = 0.357). The overall success rate was not statistically different between FOB with MA (33/36, 91.7%) and FOB alone (31/36, 86.1%); however, the cumulative success rate over time for FOB with MA was higher than that for FOB alone (*p*=0.333). For intubation using VS, there were no differences in the time to see the glottis and time to complete intubation between VS with MA and VS alone (*p*=0.065 and *p*=0.926, respectively), and the cumulative success rate was not statistically significant (*p*=0.594).

**Conclusion:**

Adjunct use of MA helped reduce time to complete intubation in FOB, but not in VS. If an inexperienced operator uses FOB, it would be helpful to use MA as an adjunct device.

## 1. Introduction

Endotracheal intubation is the most important technique in emergency airway management. Direct laryngoscope (DL), which is considered the standard method for intubation, requires the alignment of the oropharyngeal-laryngeal axis via the head tilt-chin lift maneuver to enable visualization of the glottis; therefore, intubation might be difficult if there is an anatomical abnormality or illness in that area [1]. In addition, pressure must be applied to the vallecula to raise the epiglottis to gain visibility into the glottis, and this can adversely affect the hemodynamics of the patient [2, 3]. A video laryngoscope with a similar form of DL can gain visibility into glottis with the minimal use of the head tilt-chin lift maneuver and pressure applied to the vallecula; however, it may not be completely free from causing adverse hemodynamic effects [4–7].

A fiberoptic bronchoscope (FOB) and video stylet (VS) were introduced in the 1960s and 1990s, respectively. These devices consist of a tube mounted on a video-embedded fiber or stylet and are inserted directly into the glottis; hence, there is no need to align the oropharyngeal-laryngeal axis and apply pressure to the vallecula [8–10]. For those reasons, the American Society of Anesthesiologists currently recommends FOB and VS as alternative devices for difficult airway and as rescue devices for failed airway intubation [11]. However, it may be difficult for unskilled practitioners to use these devices because the video-embedded fiber or stylet must be advanced through the pharynx and larynx into the glottis without lifting the tongue and epiglottis. For FOB, a laryngeal mask airway (LMA) or fiberoptic-compatible oral airway (FCOA) can be used as aids to create space for the fiberscope to pass through [12–14]. However, because of its nature, LMA has the disadvantage of having to be removed after the role of guiding. Commercial FCOAs are designed to pass only fibers and not the tubes; they are also to be removed after the role of guiding. For VS, no guiding device is available to date. Therefore, we designed a modified airway (MA) by creating a wide posterior channel on a preexisting Guedel-type oropharyngeal airway that could be used as a guiding device for tube insertion in both VS and FOB, which has no need to be removed after tube insertion. In this study, we aimed to determine whether our MA acts as a guide and improves the performance of intubation when used with VS or FOB for endotracheal intubation.

## 2. Materials and Methods

### 2.1. Study Design and Subjects

This randomized crossover simulation study using manikins was performed between February 2019 and May 2019 on the novice operators who had no prior experience of using VS or FOB. Participants were recruited by medical doctors who applied for the internship course at a tertiary teaching hospital in Seoul, Korea. We gained written consent from the participants after an explanation of the study brief, contents and extent of data collection, and process of the study. Anyone who did not agree to participate was excluded from the research. The study protocol was reviewed and approved by the Institutional Review Board for Human Research at Kangbook Samsung University Medical Center (approval number: IRB 2019-04-002).

### 2.2. Study Protocol

**Figure 1:**
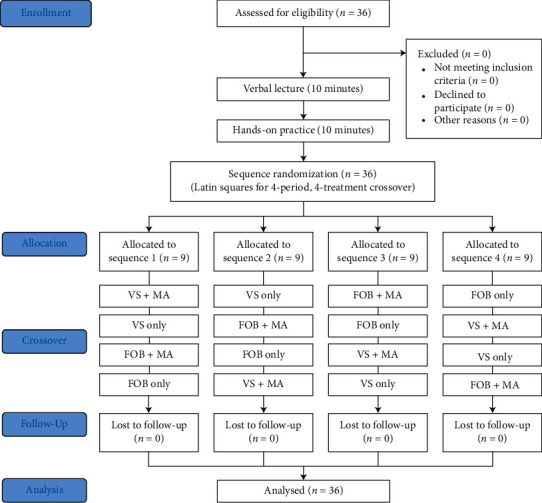
Flow diagram of the study. FOB: fiberoptic bronchoscope; VS: video stylet; MA: modified airway.

An airway management course was conducted for 20 minutes, with 10 minutes of verbal lecture and a 10-minute hands-on practice session. After the airway management course, the enrolled participants were divided into four groups by sequence randomization using Latin squares and a 4-period, 4-treatment crossover (Figure 1). We used the shuffled deck of cards method to randomly assign participants to each sequence group. The probability of random assignment to each sequence group was the same, at a 1 : 1 : 1 : 1 ratio. Participants repeated a total of four intubations under different equipment conditions. They were given more than 10 minutes of rest between each attempt.

**Figure 2:**
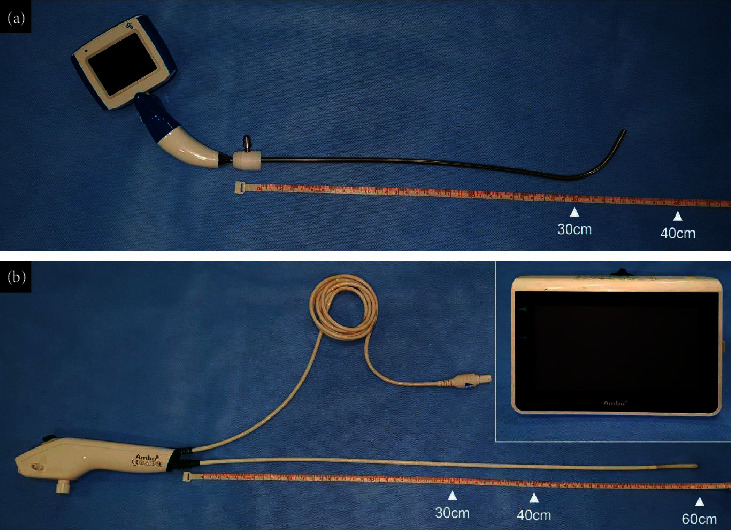
The appearance of the video stylet and the fiberoptic bronchoscope. The video stylet (VL400-S3 model from UE Medical Corp.) has a preformed semirigid stylet that is about 30 cm, almost the same as the tube, and the monitor is attached to the body of video stylet (a). The fiberoptic bronchoscope (aScopeTM 3 Regular 5.0/2.2 model from Ambu ®) has a long fiberscope that is about 60 cm, so the tube can be placed only in the proximal part of the fiberscope, and the monitor should be placed elsewhere in the table. However, the tip of the fiberscope can be manipulated by the level in the body of the fiberoptic bronchoscope (b).

**Figure 3:**
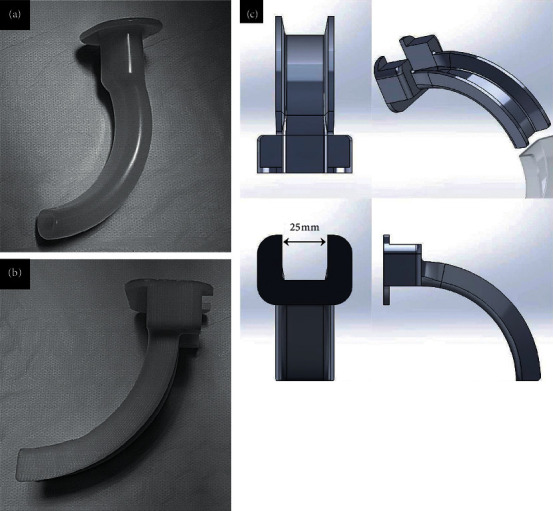
The conventional airway and the modified airway. Real photos of the conventional oropharyngeal airway (a) and the modified airway (b). 3D blueprint of the modified airway derived from conventional airway shows the 25 mm wide posterior channel (c).

The VS used was the VL400-S3 video stylet (UE Medical Corp., Zhejiang, China) (Figure 2(a)). The stylet was a preformed type measuring 30 cm long, and the monitor was attached to the body of VS. The FOB used was a ScopeTM 3 Regular 5.0/2.2 (Ambu^®^, Ballerup, Denmark) (Figure 2(b)). The length of the fiberscope was 60 cm, and the tube could only be placed in the proximal portion of the fiberscope. However, the end of the fiberscope could be manipulated by the level in the body of the FOB. The FOB monitor was placed on the table. The MA with a 25 mm wide channel on the posterior side of a preexisting Guedel-type oropharyngeal airway, which allows the tube to be inserted and released without the need for removal after intubation, was used (Figure 3).

When intubation was performed using VS, the operator mounted the tube on the stylet to make a stylet-tube assembly and advanced it along the midline of the mouth as if a curve was drawn toward the back of the tongue. When the operator recognized the glottis under the epiglottis while looking at the monitor on the body of the VS, the tube was inserted and the stylet was removed. Similar to VS, for FOB-assisted intubation, the operator mounted the tube on the fiberscope to make a fiberscope-tube assembly. Unlike VS, the fiberscope was longer than the tube; thus, the tube was mounted only on the proximal portion of the fiberscope. It was possible for the fiberscope to move upward and downward by manipulating the level in the FOB body. While manipulating the level with one hand, the operator held the fiberscope with the other and advanced it toward the glottis before inserting the tube and removing the fiberscope. The MA was then inserted between the upper and lower teeth of the manikin, and intubation with MA was performed using VS and FOB in the same way. For intubation of adults, the internal diameter (ID) of the endotracheal tube used was 7.5–9.5 mm and the outer diameter (OD) was 10.2–12.2 mm. Therefore, the diameter of the posterior channel into which the tube can be inserted and released in the MA was 25 mm. In this study, a cuffed tube with a 7.5 mm ID (OD, 10.2 mm) was used. After completing the intubation within 120 seconds, the successful intubation was confirmed upon lung expansion of the manikin.

### 2.3. Data Collection

The overall experiment was recorded using camcorders, and the intubation progress was closely recorded using cameras connected to FOB and VS. Multichannel videos were analyzed to measure and compare the success rate of intubation, time to see the glottis, and time to complete intubation of each device with or without using MA.

The success rate of intubation, time to see the glottis, and time to complete intubation of VS and FOB to the Airway Management Trainer (Laerdal, Stavanger, Norway) with or without the use of MA were measured.

The time to see the glottis was defined as the time it took for the operator to recognize the glottis from the moment the stylet or fiberscope passed through the teeth. The time to complete intubation was defined as the time at which the stylet or fiberscope was removed after inserting the tube into the glottis from the moment the stylet or fiberscope passed through the teeth. A failed intubation was defined when esophageal intubation was confirmed or the time to complete intubation exceeded 120 seconds. The time to see the glottis and time to intubation were measured for both successful and failed intubations.

### 2.4. Outcomes

The primary outcome was the time to complete intubation. The secondary outcomes were the time to see the glottis, overall success rate of intubation, and cumulative success rate of intubation.

### 2.5. Sample Size

In a pilot study using manikins, the time to complete intubation was 35.4 seconds for VS alone, 27.2 seconds for VS with MA, and 16.4 seconds for standard deviation of the difference. The time to complete intubation was 48.8 seconds for FOB alone, 36.2 seconds for FOB with MA, and 22.9 seconds for standard deviation of the difference. Assuming an alpha of 0.05, a power of 0.8, and data calculated using a two-sample paired-means test, a sample size of 34 for VS and 28 for FOB would be required to observe statistically meaningful differences.

### 2.6. Statistical Analysis

Continuous variables are presented as the medians and interquartile ranges, whereas categorical variables are presented as numbers and as frequencies. Data comparison was performed using the Wilcoxon signed-rank test or McNemar test. The effect size was calculated by dividing the *Z* statistic by the square root of the number of observations. In addition, Kaplan–Meier analysis was performed to compare the cumulative success rate over time. Kaplan–Meier plots were compared using a log-rank test. Two-sided *p* values <0.05 were considered statistically significant. Data were analyzed using statistical software (STATA 15.1 for Windows, StataCorp LLC, Texas, USA).

## 3. Results

All 36 participants were enrolled and analyzed. While all 36 participants had experience of intubating manikins using DLs, only 27.8% (10/36) had experience intubating real patients. None of the participants had experience intubating manikins or real patients using VS or FOB (Table 1).

**Figure 4:**
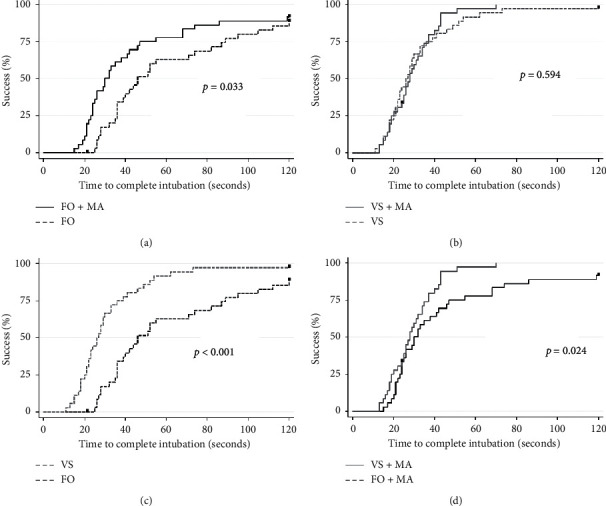
Kaplan–Meier plot of devices with or without use of modified airway. FOB: fiberoptic bronchoscope; VS: video stylet; MA: modified airway. (a) FOB + MA vs. FOB, (b) VS + MA vs. VS, (c) VS vs. FOB, and (d) VS + MA vs FOB + MA.

For intubation using FOB with MA, the median time to complete intubation was significantly reduced from 46 to 31 seconds with a medium effect size (*p*=0.004, *r* = 0.483), and the median time to see the glottis was significantly reduced from 7 to 5 seconds with a medium effect size (*p*=0.032, *r* = 0.357). The overall success rate was not statistically different between FOB with MA (33/36, 91.7%) and FOB alone (31/36, 86.1%), but the cumulative success rate over time was higher for FOB with MA than for FOB alone (*p*=0.333) (Table 2 and Figure 4).

In intubation using VS, there were no differences in the time to see the glottis and time to complete intubation between VS with MA and VS alone (*p*=0.065 and *p*=0.962, respectively), and the cumulative success rate was not statistically significant (*p*=0.594) (Table 3 and Figure 4).

When comparing VS alone and FOB alone, the median time to complete intubation was faster with VS alone (26.5 seconds) than with FOB alone (46.0 seconds) (*p* < 0.001), but the median time to see the glottis was not statistically different between VS alone (8 seconds) and FOB alone (7 seconds) (*p*=0.333). The cumulative success rate over time was higher for VS alone (35/36, 97.2%) than for FOB alone (31/36, 86.1%) (*p* < 0.001).

When comparing VS with MA and FOB with MA, the median time to complete intubation was faster using VS with MA (27.5 seconds) than using FOB with MA (31.0 seconds) (*p*=0.053), but the median time to see the glottis was not statistically significant between VS with MA (6 seconds) and FOB with MA (5 seconds) (*p*=0.042). The cumulative success rate over time was higher for VS with MA (35/36, 97.2%) than for FOB with MA (33/36, 91.7%) (*p*=0.024).

## 4. Discussion

In this study, VS with MA neither reduced the time required for intubation nor improved the success rate of intubation. These findings imply that the semirigid stylet of VS itself serves as a guide.

Unlike VS, however, FOB with MA significantly reduced the time to see the glottis and time to complete intubation, as well as the cumulative success rate. In a previous study where a skilled anesthesiologist performed an intubation using FOB in preoperative patients with normal airways, the median time to complete intubation was measured as 38.2 seconds [15]. In this study, considering that the median time to complete intubation was 46 seconds when using FOB alone and 31 seconds when using FOB with MA, it may be expected that a novice operator could intubate at the level of a skilled anesthesiologist with the assistance of MA. Using a commercial LMA or FCOA in FOB can reduce the time required for intubation [16]. However, this study is the first to reveal that the time required for intubation can be shortened even when ID of the airway is increased to 25 mm for the purpose of not to be removed after tube insertion.

A previous clinical study comparing the performance of VS and FOB alone revealed that FOB had a better success rate of intubation (VS vs. FOB and 73.3% vs. 90%), but the use of VS enabled faster completion of intubation (VS, 19.7 (19–21) seconds vs. FOB 38.2 (36–41) seconds) [15]. In contrast, in this study, compared with FOB alone, VS alone was superior with respect to the success rate of intubation and time to complete intubation. Regarding the better success rate of intubation when using FOB, a previous study reported that FOB could cope with various situations in clinical practice, such as suctioning the patient's secretions and adjusting the style angle even after passing through the mouth, which cannot be done with VS. However, because the previous study was conducted with experienced anesthesiologists, the experience and instrumental dexterity of the practitioner could be a source of bias. In comparison, this study was conducted with novice operators who had no experience of using VS and FOB; hence, there was no handling bias, and the performance of the devices could be more accurately measured. The results of this study indicate that VS rather than FOB is better for novice operators with an improvement in the intubation success rates and shorter times to complete intubation. The reason for these results seems to be that VS is simpler and more intuitive to operate than FOB, although the visibility of the VS and FOB are similar.

As FOB began to be used as intubation equipment about 30 years before VS, studies on the necessity and effectiveness of adjunct devices, such as FCOA, were conducted [8, 12]. However, because VS appeared belatedly, there are not many related studies and no related commercial products on the adjunctive device. The MA used by the authors in this study was modified to be used on both FOB and VS. This MA is similar in appearance to Luomanen or Ovassapian airway with the posterior channel, but there are some differences [12]. First, since the posterior channel of MA is wider than the existing FCOA's, an ET tube can be entered and removed through this widened space, and a suction tube can be inserted if necessary. Second, the channel of MA extends to the tip. This is the same as Luomanen, but different from the Ovassappian airway. Since the channel extends to the tip, it is an advantage not to deviate from the midline unnecessarily, but it is a disadvantage not to be able to freely explore the surrounding structures beyond the midline.

This study had some limitations. First, this study was a simulation study using manikins and was not conducted in the real clinical environment. Situations in which suction was necessary and inability to head tilt due to poor neck mobility or cervical spine injury were not considered. If MA is studied in the actual clinical situation, it should be taken into account not only the issue of securing vision but also the positive effect of easily suctioning the secretion. This is because it is possible to immediately intubate without removing the MA after the suctioning procedure.

Second, this study was conducted at a single center. In order to generalize, further studies are needed on real patients in various clinical situations. Third, participants in this study were not experienced practitioners. Although this allowed us to focus on the performance of the device itself, it is possible that the results may differ from those of expert practitioners because intubation may vary with an individual's skill levels. However, in reality, equipment such as FBO and VS are not the primary equipment for emergency airway intubation, so even physicians who are experienced with a direct laryngoscope or video stylet do not have much experience in using this equipment.

Finally, in this manikin study, adjunct use of MA helped reduce the time to complete intubation in FOB, but not in VS. If an inexperienced operator uses FOB, it would be helpful to use MA as an adjunct device.

## Figures and Tables

**Table 1:** Demographic data and intubation experience of participants with respect to the types of devices used for manikins or real patients.

Variable	Median (IQR)
Male, *n* (%)	22 (61.11%)
Female, *n* (%)	14 (38.89%)
Age, years	26 (25, 27)
Height, cm	172 (161, 176.5)
Weight, kg	66 (52, 74.5)
Experience of intubation by DL to manikin, *n* (%)	36 (100%)
Experience of intubation by DL to real patient, *n* (%)	10 (27.78%)
Experience of intubation by FOB or VS, *n* (%)	0 (0%)

DL, direct laryngoscope; FOB, fiberoptic bronchoscope; VS, video stylet.

**Table 2:** Comparison of fiberoptic bronchoscope with modified airway and fiberoptic bronchoscope alone.

	FOB with MA	FOB alone	*p* value	Effect size (*r*)
Time to see the glottis, seconds	5 (4, 7.5)	7 (5, 10)	0.032	0.357
Time to complete intubation, seconds	31 (23.5, 51)	46 (35.5, 88)	0.004	0.483
Success, *n* (%)	33 (91.67%)	31 (86.11%)	0.710	—

FOB, fiberoptic bronchoscope; MA, modified airway.

**Table 3:** Comparison of video stylet with modified airway and video stylet alone.

	VS with MA	VS only	*p* value	Effect size (*r*)
Time to see the glottis, seconds	6 (4, 9)	8 (5, 18)	0.065	0.312
Time to complete intubation, seconds	27.5 (19.5, 36)	26.5 (20.5, 37.5)	0.962	0.008
Success, *n* (%)	35 (97.22%)	35 (97.22%)	1.000	—

VS, video stylet; MA, modified airway.

## Data Availability

The data are available from the corresponding author on reasonable request.
